# Closed Incision Negative Pressure Therapy for the Recipient Site in Head and Neck Reconstruction

**DOI:** 10.1002/oto2.70249

**Published:** 2026-05-22

**Authors:** Haruyuki Hirayama, Katsuhiro Ishida, Hiroki Kodama, Doruk Orgun, Masaki Nukami, Masato Nagaoka, Taisuke Akutsu, Soichiro Fukuzato, Takeshi Miyawaki

**Affiliations:** ^1^ Department of Plastic and Reconstructive Surgery The Jikei University School of Medicine Tokyo Japan; ^2^ Department of Otorhinolaryngology The Jikei University School of Medicine Tokyo Japan

**Keywords:** closed incision negative pressure therapy, head and neck reconstruction, postoperative complication, reconstructive surgery, surgical site infection

## Abstract

**Objective:**

This study aimed to evaluate the benefits of recipient site of closed incision negative pressure therapy (ciNPT) on postoperative complications in head and neck reconstruction.

**Study Design:**

A single‐center retrospective observational study was conducted on patients who underwent head and neck reconstruction for both benign and malignant disease using pedicled or free tissue transfers between November 2023 and September 2024.

**Setting:**

A tertiary care institution.

**Methods:**

Patients were divided into ciNPT and non‐ciNPT groups. 30‐day postoperative complication rates, including skin necrosis, surgical site infection (SSI), hematoma, lymphorrhea, anastomotic leakage, and flap vascular compromise, were compared between groups.

**Results:**

44 patients (median age: 68.0, female: 38.6%) were included in the analyses (21 ciNPT, 23 non‐ciNPT). The ciNPT group had a higher median intraoperative blood loss (510 vs 175 mL; *P* = .004) and higher rates of intra/postoperative blood transfusion (5 vs 0; *P *= .019). A total of 39 patients underwent free tissue transfer, comprising 17 in the ciNPT group (81.0%) and 22 in the non‐ciNPT group (95.7%). Clavien‐Dindo grade IIIa complications occurred in 19.0% of ciNPT and 21.7% of non‐ciNPT cases (*P* = 1.000), while grade IIIb complications occurred in 19.0% and 13.0%, respectively (*P* = .693). Overall, no significant differences were found between groups across complication categories.

Although overall complication rates were comparable between the 2 groups, this study emphasizes practical techniques for the clinical application of ciNPT in complex reconstruction cases. Larger, prospective, multi‐center studies are warranted to validate its benefits and optimize clinical use.

**Level of Evidence:**

Ⅲ.

Negative pressure wound therapy (NPWT) has become widespread in clinical practice since Argenta et al reported its first use on acute and chronic wounds in 1997.^1^ A variant of NPWT, the closed incision negative pressure therapy (ciNPT), has been developed for postoperative surgical incision management and has increasingly been adopted across various surgical fields. In plastic and reconstructive surgery, the application of ciNPT has been reported in procedures such as breast reconstruction, decubitus ulcer reconstruction, abdominoplasty, ventral hernia repair, sternal wound reconstruction, and inguinal lymph node dissection.^2^, ^3^, ^4^, ^5^, ^6^, ^7^, ^8^, ^9^, ^10^, ^11^, ^12^, ^13^, ^14^ However, reports on the use of ciNPT in head and neck reconstruction are limited, and the only available studies address its utilization on donor sites.^15^, ^16^ One such study investigated its application to scapula and latissimus dorsi harvest sites, but failed to demonstrate a reduction of major wound site complications with the use of NPWT.^15^ Another study reported associations between ciNPT use and the reduced risk of donor‐site complications in head and neck reconstruction.^16^ Postoperative complications following head and neck cancer surgery are associated with hospital stay durations, mortality rates, and treatment costs.^17^ Therefore, minimizing the occurrence of complications is a crucial concern.

We hypothesized that the use of ciNPT is associated with a reduced risk of postoperative complications at the recipient site in head and neck reconstruction with tissue transfers. To evaluate this hypothesis, we compared 30‐day postoperative complication rates, types of complications, and severity between cases with and without ciNPT application.

## Materials and Methods

### CiNPT Management Technique

The application of ciNPT to the head and neck is more challenging in maintaining an airtight seal and applying negative pressure compared to flat wound surfaces due to the presence of anatomical structures such as the mandible, clavicle, and auricle, as well as surgically created features including a tracheostomy stoma, a monitoring skin paddle, and a monitoring jejunum.

**Figure 1:**
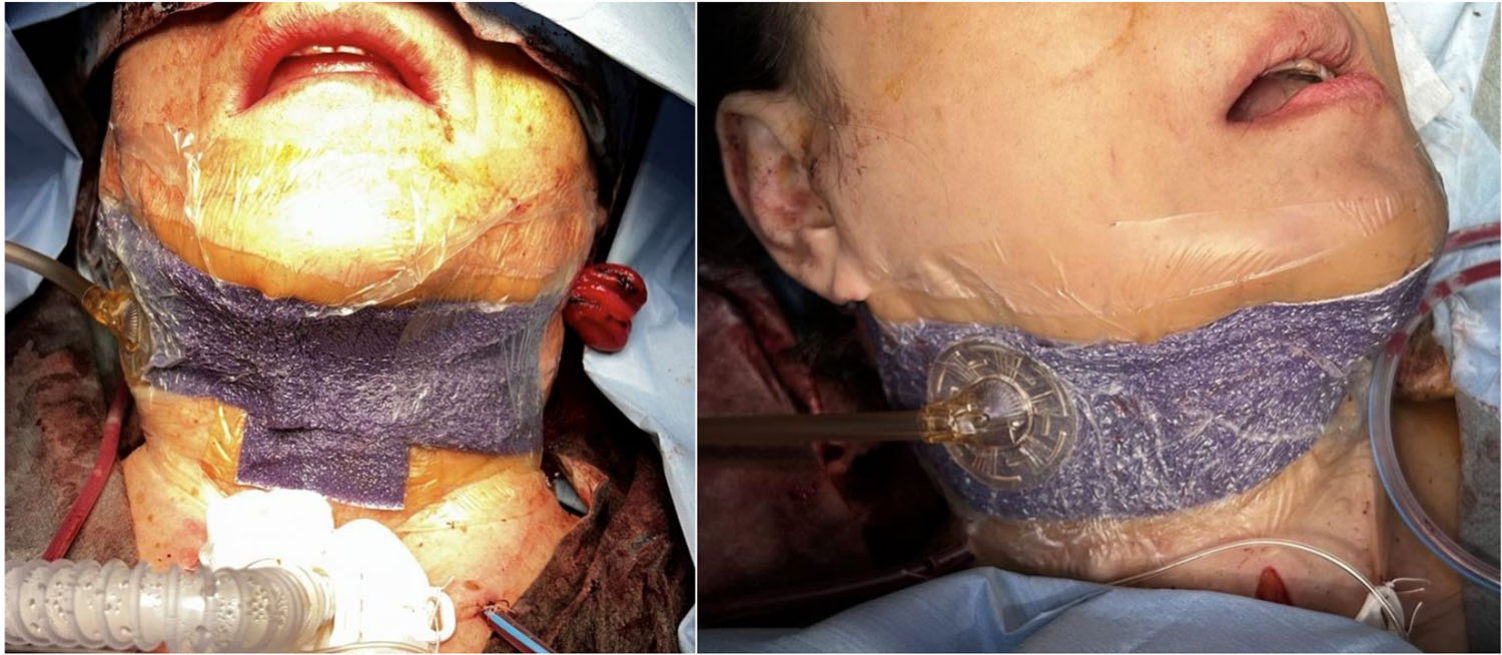
Closed incision negative pressure therapy was performed after head and neck reconstruction.

The 3 M™ Prevena™ Plus Customizable Incision Management System (KCI, part of 3 M Company) was applied to a wound in the head and neck region (Figure 1). This product has incisions in the foam dressing, allowing it to flex in multiple directions and adapt to nonflat wound surfaces in the head and neck region.

Before dressing application, the periwound skin was cleaned, degreased, and thoroughly dried to maximize adhesion of the film drape. The customizable foam was trimmed and/or segmented to accommodate contour changes. The film drape was applied over the foam to achieve an airtight seal. In areas where film dressing is difficult to adhere to the skin due to anatomical irregularities such as the clavicle and mandible, securing the film with a skin stapler is recommended. Additionally, in regions near the oral cavity and cranial side of tracheostomy stoma, where the film dressing is prone to detachment due to saliva/sputum, applying a hydrocolloid wound dressing to the skin before placing the film dressing can enhance adhesion. When the monitoring skin paddle or jejunum is positioned in the neck for postoperative flap perfusion assessment, continuous observation is necessary. To facilitate this, foam dressing should be applied around the edges of the monitoring skin paddle, leaving its center visible, or alternatively, these areas can be avoided by bridging the foam dressing. For other wound areas, it is recommended to cover them with foam dressing as much as possible. Closed suction drains were placed for several days postoperatively in most cases. However, to minimize the negative pressure impact exerted by the drain on the pedicle, it is essential to position the drain at a site sufficiently distant from the pedicle. Based on our experience, we consider the application of ciNPT to be unfeasible in cases of external auditory canal or parotid gland cancer, where the wound extends around the auricle.

ciNPT was generally maintained until postoperative day (POD) 7. During ciNPT application, if an air leak occurred, the seal was reinforced with film dressing or hydrocolloid wound dressing as needed to ensure the maintenance of negative pressure.

### Study Population

This study was a retrospective observational study of patients who underwent head and neck reconstruction at a single institution (The Jikei University Hospital) between November 2023 and September 2024. Incidences of postoperative complications in the head and neck were compared between groups where ciNPT was or was not utilized. The decision to apply ciNPT was made at the discretion of the attending reconstructive surgeon, taking into account factors such as wound tension, anticipated dead space, prior radiation exposure, and the complexity of the reconstruction, as part of routine clinical practice. All procedures performed in this study involving human participants were conducted in accordance with the ethical standards of the institutional and/or national research committee and with the 2002 version of the World Medical Association Declaration of Helsinki. This study was independently reviewed and approved by the Ethics Committee of the Jikei University School of Medicine (approval number 36‐194 (12303)). We used an opt‐out approach, and none of the patients refused to consent.

The inclusion criterion was head and neck reconstruction where flap transfer methods were utilized. The exclusion criteria were as the following: cases with lesions located in the maxilla, external auditory canal, and parotid, cases of pharyngocutaneous fistulae following total laryngectomy, cases that were deemed by the investigators to be unfeasible for participation in this study, and cases where patients refused to participate in the study.

### Outcomes

The primary endpoint was the occurrence of postoperative complications in the head and neck within 30 days after surgery. Postoperative complications in the head and neck were defined as skin necrosis, surgical site infection (SSI), hematoma, lymphorrhea, anastomotic leakage, flap vascular compromise (Clavien‐Dindo (CD) classification^18^ grade [Table 1] Ⅲa or higher). The criteria of the Centers for Disease Control and Prevention (CDC) were adopted for SSI.^19^ The definitions of hematoma and lymphorrhea in this study were those diagnosed by properties of fluids when there was swelling and puncture or opening the wound or when there was wound dehiscence. Flap monitoring was performed using clinical assessment of the skin paddle, including visual inspection and pinprick testing. No handheld or implantable Doppler devices were used. The same monitoring protocol was applied consistently to both the ciNPT and non‐ciNPT groups. The secondary endpoints were the occurrence of distinct types of complications including skin necrosis, surgical site infection (SSI), hematoma, lymphorrhea, anastomotic leakage, and flap vascular compromise.

**Table 1:** Summary of the Clavien–Dindo Classification Used in This Study

Grade	Definition
Grade I	Minor deviation from the expected postoperative course not requiring pharmacological therapy beyond routine supportive medications or any invasive intervention. Permitted treatments include antiemetics, antipyretics, analgesics, diuretics, electrolyte replacement, physiotherapy, and bedside opening of superficial wound infections
Grade II	Complications requiring pharmacologic therapy beyond routine supportive treatment, including blood transfusion or total parenteral nutrition
Grade III	Complications requiring procedural intervention
Grade IIIa	Interventions not requiring general anesthesia
Grade IIIb	Interventions requiring administration of general anesthesia
Grade IV	Severe complications posing an immediate threat to life and necessitating management in an intensive care setting
Grade IV	Failure of a single organ system (including need for dialysis)
Grade IVb	Failure involving multiple organ systems
Grade V	Patient mortality

Complications were classified according to the Clavien–Dindo system (Dindo et al, 2004).

### Patient Characteristics

Patient characteristics included age, sex, body mass index (BMI), hypertension (HT), diabetes mellitus (DM), chronic obstructive pulmonary disease (COPD), steroid/immunosuppressant use, smoking, preoperative chemotherapy, preoperative radiotherapy, history of head and neck surgery, neck dissection, bony reconstruction, American Society of Anesthesiologists physical status (ASA‐PS),^20^ preoperative serum albumin level, preoperative serum hemoglobin level, operating time, blood loss, blood transfusion, lesion site, pT and pN category, surgical wound classification, flap type, perioperative prophylactic antibiotic, duration of prophylactic antibiotic administration and duration of intensive care unit stay.

### Statistical Analysis

We used Fisher's exact test for categorical variables between 2 groups, while applying Mann‐Whitney *U* test to compare continuous variables. All *P *< .05 were considered as statistically significant.

## Results

Of the 73 eligible cases where flap harvest sites were examined, we excluded the following: 17 cases with maxillary lesions, 3 cases with external auditory canal lesions, 7 cases with parotid gland lesions, and 2 cases of pharyngocutaneous fistulae following total laryngectomy. As a result, 44 cases were included in the analysis, of whom 21 (47.7%) utilized ciNPT.

By postoperative day (POD) 7, a total of 5 cases experienced ciNPT air leakage. In 2 of these cases, reinforcement allowed ciNPT to be reapplied and continued until POD7, whereas in the other 3 cases, air leakage occurred on POD6, leading to premature termination of ciNPT before POD7. In 2 cases, ciNPT was discontinued on POD1 and POD2, respectively, due to flap vascular compromise. Ultimately, ciNPT was applied until POD7 in 16 of the 21 cases.

### Patient Characteristics

Comparison of patient characteristics between the ciNPT and non‐ciNPT groups are shown in Table 2.

**Table 2:** Comparison of Patient Characteristics Between the ciNPT and Non‐ciNPT Groups

	CiNPT	Non‐ciNPT	*P* value
No. of cases (%)	21 (47.7)	23 (52.3)	
Age (year), median (IQR)	64.0 (56.0‐74.5)	71.0 (55.0‐76.0)	.564^a^
Sex, n (%)			
Male (n = 27)	13 (61.9)	14 (60.9)	1.000^b^
Female (n = 17)	8 (38.1)	9 (39.1)	
BMI (kg/m^2^), median (IQR)	22.9 (18.7‐25.3)	21.4 (19.9‐24.4)	.605^a^
HT, n (%)			
Yes (n = 11)	5 (23.8)	6 (26.1)	1.000^b^
No (n = 33)	16 (76.2)	17 (73.9)	
DM, n (%)			
Yes (n = 7)	5 (23.8)	2 (8.7)	.232^b^
No (n = 37)	16 (76.2)	21 (91.3)	
COPD, n (%)			
Yes (n = 0)	0 (0.0)	0 (0.0)	1.000^b^
No (n = 44)	21 (100.0)	23 (100.0)	
Steroid/immunosuppressant use, n (%)			
Yes (n = 4)	2 (9.5)	2 (8.7)	1.000^b^
No (n = 40)	19 (90.5)	21 (91.3)	
Smoking, n (%)			
Never smoked (n = 15)	6 (28.6)	9 (39.1)	.102^b^
Former smoker (n = 11)	3 (14.3)	8 (34.8)	
Active smoker (n = 18)	12 (57.1)	6 (26.1)	
Preoperative chemotherapy, n (%)			
Yes (n = 8)	5 (23.8)	3 (13.0)	.449^b^
No (n = 36)	16 (76.2)	20 (87.0)	
Preoperative radiotherapy, n (%)			
Yes (n = 8)	4 (19.0)	4 (17.4)	1.000^b^
No (n = 36)	17 (81.0)	19 (82.6)	
History of head and neck surgery, n (%)			
Yes (n = 16)	7 (33.3)	9 (39.1)	.761^b^
No (n = 28)	14 (66.7)	14 (60.9)	
Neck dissection, n (%)			
Bilateral (n = 14)	7 (33.3)	7 (30.4)	.864^b^
Unilateral (n = 12)	5 (23.8)	7 (30.4)	
None (n = 18)	9 (42.9)	9 (39.1)	
Bony reconstruction, n (%)			
Yes (n = 18)	10 (47.6)	8 (34.8)	.541^b^
No (n = 26)	11 (52.4)	15 (65.2)	
ASA‐PS, n (%)			
Ⅰ (n = 9)	3 (14.3)	6 (26.1)	.296^b^
Ⅱ (n = 33)	16 (76.2)	17 (73.9)	
Ⅲ (n = 2)	2 (9.5)	0 (0.0)	
Ⅳ (n = 0)	0 (0.0)	0 (0.0)	
Ⅴ (n = 0)	0 (0.0)	0 (0.0)	
Ⅵ (n = 0)	0 (0.0)	0 (0.0)	
Preoperative serum albumin level (g/dL), median (IQR)	3.9 (3.3‐4.4)	4.2 (4.0‐4.5)	.101^a^
Preoperative serum hemoglobin level (g/dL), median (IQR)	12.5 (9.6‐13.8)	12.9 (12.2‐14.4)	.133^a^
Operating time (min), median (IQR)	490 (361‐561)	459 (351‐542)	.466^a^
Blood loss (mL), median (IQR)	510 (185‐655)	175 (110‐420)	.004^a^
Blood transfusion, n (%)			
Yes (n = 5)	5 (23.8)	0 (0.0)	.019^b^
No (n = 39)	16 (76.2)	23 (100.0)	
Lesion site, n (%)			
Oral cavity (n = 13)	6 (28.6)	7 (30.4)	.286^b^
Oropharynx (n = 4)	0 (0.0)	4 (17.4)	
Hypopharynx (n = 10)	5 (23.8)	5 (21.7)	
Larynx (n = 1)	1 (4.8)	0 (0.0)	
Mandible (n = 16)	9 (42.9)	7 (30.4)	
pT category, n (%)			
T0 (n = 16)	7 (33.3)	9 (39.1)	.064^b^
T1 (n = 0)	0 (0.0)	0 (0.0)	
T2 (n = 4)	1 (4.8)	3 (13.0)	
T3 (n = 11)	3 (14.3)	8 (34.8)	
T4 (n = 13)	10 (47.6)	3 (13.0)	
pN category, n (%)			
N0 (n = 27)	12 (57.1)	15 (65.2)	.303^b^
N1 (n = 5)	1 (4.8)	4 (17.4)	
N2 (n = 4)	2 (9.5)	2 (8.7)	
N3 (n = 8)	6 (28.6)	2 (8.7)	
Surgical wound classification, n (%)			
Class Ⅰ/Clean (n = 0)	0 (0.0)	0 (0.0)	1.000^b^
ClassⅡ/Clean‐contaminated (n = 34)	16 (76.2)	18 (78.3)	
Class Ⅲ/Contaminated (n = 0)	0 (0.0)	0 (0.0)	
Class Ⅳ/Dirty‐infected (n = 10)	5 (23.8)	5 (21.7)	
Flap type, n (%)			
PMMC (n = 2)	2 (9.5)	0 (0.0)	.019^b^
Scapular (n = 3)	1 (4.8)	2 (8.7)	
LD (n = 0)	0 (0.0)	0 (0.0)	
RF (n = 0)	0 (0.0)	0 (0.0)	
RAMC (n = 0)	0 (0.0)	0 (0.0)	
DIEP (n = 6)	0 (0.0)	6 (26.1)	
FJ (n = 6)	5 (23.8)	1 (4.3)	
ALT (n = 12)	4 (19.0)	8 (34.8)	
Fibular (n = 10)	5 (23.8)	5 (21.7)	
Fibular and another (n = 5)	4 (19.0)	1 (4.3)	
Pedicled or free flap, n (%)			
Pedicled (n = 3)	2 (9.5)	1 (4.3)	.310^b^
Free (n = 39)	17 (81.0)	22 (95.7)	
Both (n = 2)	2 (9.5)	0 (0.0)	
Prophylactic antibiotic, n (%)			
Ampicillin/sulbactam (n = 37)	17 (81.0)	20 (87.0)	.693^b^
Non‐ampicillin/sulbactam (n = 7)	4 (19.0)	3 (13.0)	
Duration of prophylactic antibiotic administration (days), median (IQR)	9.0 (7.0‐17.5)	8.0 (6.0‐15.0)	.408^a^
Duration of intensive care unit stay (days), median (IQR)	1.0 (1.0‐1.0)	1.0 (1.0‐1.0)	.505^a^

Percentages may not equal 100% due to rounding.

Abbreviations: ALT, anterolateral thigh; ASA‐PS, American Society of Anesthesiologists physical status; BMI, body mass index (weight [kg]/height squared [m2]); CiNPT, closed incision Negative Pressure Therapy; COPD, chronic obstructive pulmonary disease; DIEP, deep inferior epigastric perforator; DM, diabetes mellitus; FJ, free jejunal; HT, hypertension; IQR, interquartile range; LD, latissimus dorsi; PMMC, pectoralis major myocutaneous; pN, pathological Node; pT, pathological Tumor; RAMC, rectus abdominis musculocutaneous; RF, radial forearm.

^a^ *P* values were calculated using Mann‐Whitney *U* test for continuous variables. Statistical significance was set at *α* = .05.

^b^ *P* values were calculated using Fisher's exact test for categorical variables. Statistical significance was set at 2‐tailed *α* = .05.

The ciNPT group had greater median blood loss (510 vs 175 mL, *P* = .004). Additionally, blood transfusions were more frequent in the ciNPT group compared to the non‐ciNPT group (23.8% vs 0%, *P* = .019).

### Head and Neck Complications

The comparison of head and neck complications (CD Classification IIIa) between the ciNPT and non‐ciNPT groups is shown in Table 3. Overall complications classified as CD grade IIIa were observed in 19.0% of cases in the ciNPT group and 21.7% in the non‐ciNPT group (*P* = 1.000). No significant differences in the incidence of each complication were observed between the 2 groups.

**Table 3:** Comparison of Each Head and Neck Complication (CD Classification Ⅲa) Between the ciNPT and Non‐ciNPT Groups

CD classification Ⅲa	CiNPT (n = 21)	Non‐ciNPT (n = 23)	*P* value
Overall complications, (%)	4 (19.0)	5 (21.7)	1.000
Skin necrosis, n (%)	1 (4.8)	1 (4.3)	1.000
SSI, n (%)	1 (4.8)	1 (4.3)	1.000
Hematoma, n (%)	1 (4.8)	0 (0.0)	.477
Lymphorrhea, n (%)	0 (0.0)	1 (4.3)	1.000
Anastomotic leakage, n (%)	1 (4.8)	0 (0.0)	.477
Flap vascular compromise, n (%)	1 (4.8)	2 (8.7)	1.000

Percentages may not equal 100% due to rounding. P values were calculated using Fisher's exact test for categorical variables. Statistical significance was set at 2‐tailed *α* = .05.

Abbreviations: CD, Clavien‐Dindo; CiNPT, closed incision Negative Pressure Therapy; SSI, surgical site infection.

The comparison of head and neck complications (CD Classification IIIb) between the ciNPT and non‐ciNPT groups is presented in Table 4. Overall complications occurred in 19.0% of cases in the ciNPT group and 13.0% in the non‐ciNPT group (*P* = .693). There was no statistically significant difference in the distribution of these complications between the 2 groups.

**Table 4:** Comparison of Each Head and Neck Complication (CD Classification Ⅲb) Between the ciNPT and Non‐ciNPT Groups

CD classification Ⅲb	CiNPT (n = 21)	Non‐ciNPT (n = 23)	*P* value
Overall complications, (%)	4 (19.0)	3 (13.0)	.693
Skin necrosis, n (%)	1 (4.8)	0 (0.0)	.477
SSI, n (%)	0 (0.0)	0 (0.0)	1.000
Hematoma, n (%)	0 (0.0)	2 (8.7)	.489
Lymphorrhea, n (%)	0 (0.0)	0 (0.0)	1.000
Anastomotic leakage, n (%)	0 (0.0)	0 (0.0)	1.000
Flap vascular compromise, n (%)	3 (14.3)	1 (4.3)	.335

Percentages may not equal 100% due to rounding.

Abbreviations: CD, Clavien‐Dindo; CiNPT, closed incision Negative Pressure Therapy; SSI, surgical site infection.

### Causes and Recipient Site Vessels in Cases of Flap Vascular Compromise

Regarding flap vascular compromise, the CD grade IIIa cases all presented with partial flap necrosis and the underlying cause remained unclear. Therefore, only the CD grade IIIb cases were analyzed in greater detail (Table 5).

**Table 5:** Causes and Recipient Site Vessels in Cases of Flap Vascular Compromise (CD Classification Ⅲb)

Lesion site	Flap type	Recipient site artery	Recipient site vein	Cause	Anastomotic thrombosis	Pedicle torsion
*CiNPT group*						
Mandible	Fibular	Superior thyroid artery	Facial vein	Congestion	No	No
Mandible	ALT (and fibular)	Distal fibular artery	Distal fibular vein	Ischemia (ALT)	No	No
Mandible	Fibular(and ALT)	Superior thyroid artery(vein graft)	External jugular vein(vein graft)	Ischemia (Fibular)	Yes	No
*Non‐ciNPT group*						
Oropharynx	ALT	Superior thyroid artery	Internal jugular vein	Congestion	No	Yes

Abbreviations: ALT, anterolateral thigh; CD, Clavien‐Dindo; CiNPT, closed incision Negative Pressure Therapy.

In the ciNPT group, three cases of flap vascular compromise classified as CD grade IIIb were reported. The first case involved a fibular flap used for mandibular reconstruction. The superior thyroid artery served as the recipient artery, while the facial vein was utilized as the recipient vein. The complication observed was venous congestion on POD2, with no evidence of anastomotic thrombosis or pedicle torsion. The second case involved a combined ALT and fibular flaps used for mandibular reconstruction. In this case, the distal fibular artery and vein were used as recipient vessels for the ALT flap. The underlying cause of the ALT flap's vascular compromise was ischemia on POD1, with no anastomotic thrombosis or pedicle torsion observed. In the third case, mandibular reconstruction was carried out using a combination of ALT and fibular flaps. The recipient vessels for the fibular flap were the superior thyroid artery and the external jugular vein, both anastomosed via vein grafts. On POD1, ischemia occurred, and re‐exploration revealed thrombosis at the anastomotic site between the superior thyroid artery and the vein graft.

In the non‐ciNPT group, one case of flap vascular compromise classified as CD grade IIIb was documented. This case involved an ALT flap used for oropharyngeal reconstruction. The superior thyroid artery served as the recipient artery, while the internal jugular vein was used as the recipient vein. The primary cause of vascular compromise in this case was venous congestion, and pedicle torsion was identified as a contributing factor. No anastomotic thrombosis was observed in this case. The congestion resolved after the release of pedicle torsion.

## Discussion

In this study of patients undergoing head and neck reconstruction, we compared the complication profiles of patients that received ciNPT at the recipient site with those who did not. Incidences of head and neck complications were similar between groups for both CD classification IIIa and IIIb. It is important to emphasize the difference in baseline characteristics between the two groups. As such, patients in the ciNPT group had significantly greater blood loss and a higher requirement for transfusion compared to those in the non‐ciNPT group. This difference may reflect the fact that more complex reconstructions were performed in the ciNPT group, as indicated by the distribution of flap types. For example, combined fibular flaps, which are generally associated with high intraoperative blood loss, were more common in the ciNPT group, whereas ALT and DIEP flaps predominated in the non‐ciNPT group. This imbalance suggests the possibility of selection bias, where ciNPT was more likely to be applied in higher‐risk or technically demanding reconstructions. Importantly, despite the higher blood loss and the predominance of more complex flap types in the ciNPT group, the incidence of postoperative complications did not differ significantly between the two groups. This finding highlights the potential benefit of ciNPT in mitigating wound‐related risks even in patients undergoing more invasive reconstructions.

**Figure 2:**
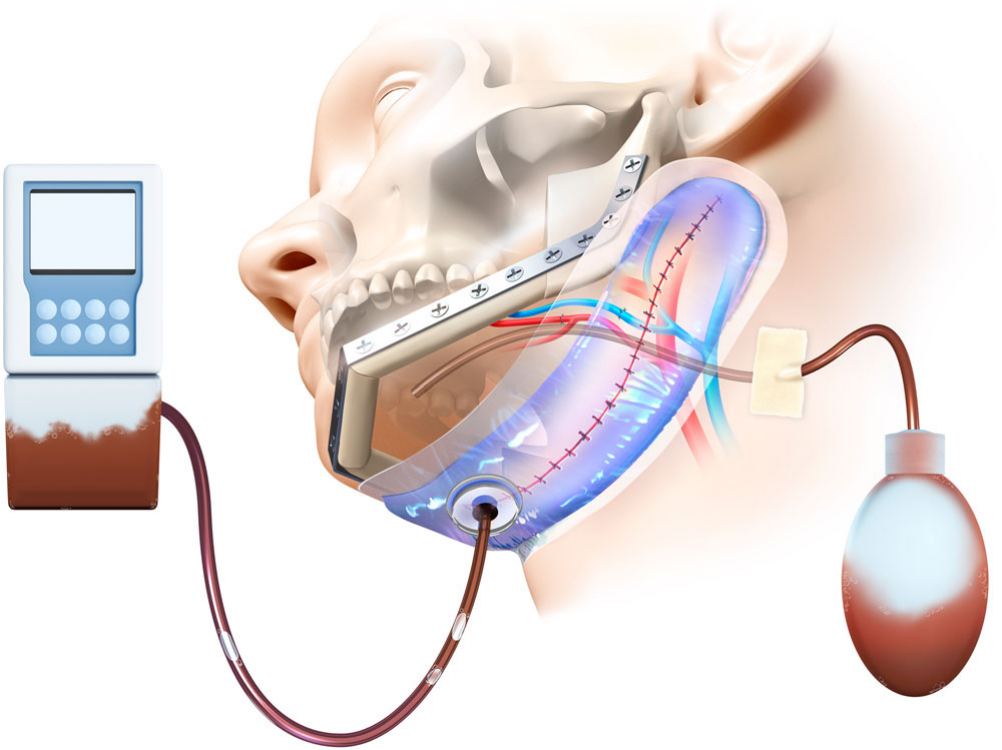
Schematic illustration showing the application of closed incision negative pressure therapy to the head and neck region following reconstructive surgery.

ciNPT has benefits on wound healing by: acting as a barrier to external contamination,^21^ helping to hold the incision edges together,^22^ decreasing lateral tension of sutured/stapled incisions,^23^ removing fluids and infectious materials and reducing edema.^24^ Due to the three‐dimensional complex structure created by the mandible and the clavicle, dead space is often formed after head and neck surgery and can contribute to the development of SSI, hematoma, and lymphorrhea. The use of ciNPT may help reduce these complications by decreasing dead space through negative pressure (Figure 2). As such, one meta‐analysis demonstrated that ciNPT performed significantly better at reducing the incidence of SSIs than traditional dressings in various surgical fields.^25^

Furthermore, in this study, we investigated the causes and recipient site vessels in three cases of flap vascular compromise (3 in the ciNPT group and 1 in the non‐ciNPT group). In the non‐ciNPT group, pedicle torsion caused flap congestion, which resolved after reopening the wound and releasing the torsion. On the other hand, in 2 cases within the ciNPT group, neither anastomotic thrombosis nor pedicle torsion was the cause of flap vascular compromise. One case in the ciNPT group where the facial vein was selected as the recipient vein, resulted in flap congestion. Although ciNPT had been removed at the time of reoperation, the negative pressure might have caused the collapse of the thin‐walled facial vein between the overlying skin and the underlying tissue. Some studies have reported that NPWT dressings lead to an increase in underlying pressure. However, while significant pressure increases were observed at a distance of 1 cm from the NPWT, the changes at 2 and 3 cm were minimal.^26^ It is therefore necessary to carefully select the application site of ciNPT to ensure that the pedicle does not pass within a depth of 1 cm from the foam dressing. In one other case of ischemia in the ciNPT group where reconstruction was performed using double free flaps, the distal portion of one of the flap pedicles was selected as the recipient artery. Here, the negative pressure of ciNPT could have contributed to kinking of the flap vessels, which may have had reduced flexibility due to preexisting arterial sclerosis. However, a direct causal relationship between ciNPT application and flap vascular compromise cannot be established due to the retrospective design and potential selection bias. Nevertheless, external pressure from the device remains a plausible, albeit unproven, mechanism for the observed complications in these cases. When using ciNPT, caution may be required in cases where the recipient vein or the vein of the pedicle is located just beneath the skin, as well as when utilizing lower limb flaps in elderly patients who may have arterial sclerosis. Additionally, in many head and neck surgeries, the procedure is performed with the neck in an extended position. If ciNPT is applied during neck extension, the pedicle position may shift when neck extension is released. Therefore, it is recommended to apply ciNPT after the patient has been positioned in their postoperative posture. Moreover, closed suction drains are frequently placed intraoperatively, exerting additional negative pressure. Therefore, when using ciNPT, meticulous attention should be given to the placement of the drain to minimize the risk of complications. The adjunctive use of an intraluminal monitoring device may be beneficial for the objective confirmation of vascular patency.^27^

In this study, ciNPT was performed using a device that applied negative pressure at −125 mmHg; however, it remains unclear whether −125 mmHg is the optimal pressure. Studies utilizing ciNPT at a negative pressure of −80 mmHg have also been reported.^28^, ^29^, ^30^, ^31^, ^32^, ^33^ A meta‐analysis of total hip and knee arthroplasties indicated that patients undergoing treatment with ciNPT suggested that pressures of −125 mmHg might reduce infection risk.^34^ Moreover, we applied ciNPT for 7 days, based on the manufacturer's recommendation. To the best of our knowledge, no studies have compared different durations of ciNPT, and further investigations are necessary.

Concerns regarding using ciNPT in head and neck reconstruction include surgical site observation and cost. Although it is important to observe the surgical site and respond promptly when complications are suspected; the clinician can in change utilize temperature monitoring, blood tests, and imaging studies. Papp et al reported that ciNPT reduces postoperative complications, shortens hospital length of stay, and reduces the number of recurrent open wounds at 3 months after pressure ulcer reconstruction in patients with spinal cord impairment, resulting in significant cost savings.^6^ Munro et al reported that the use of ciNPT at DIEP flap donor sites resulted in a statistically significant improvement in complication‐associated costs of £420.77.^35^ Several other studies have also stated that ciNPT is cost‐effective.^36^, ^37^, ^38^

Furthermore, there is evidence that ciNPT can help reduce flap edema and support free cutaneous flaps by this phenomenon. In a study by Muller‐Sloof et al., no splints were necessary in extremity reconstruction cases due to the relative immobilizing effect of the negative pressure device.^39^ In head and neck reconstruction recipient sites, the relative immobilizing effect may stabilize the postoperative wound and potentially reduce complications such as hematoma. However, since its application can be somewhat challenging and that air leakage may occur and require management, the development of a device that can be applied more easily and safely to the head and neck is warranted.

### Limitations

The single‐center retrospective design of the study risks selection bias and residual confounding since ciNPT use was not randomized. Baseline characteristics also differed between groups (eg, greater blood loss and more complex reconstructions in the ciNPT group), and the inclusion of different flap types and a mix of benign and malignant primary diseases may have influenced the results, limiting direct comparability even after groupwise analyses.

We assessed multiple endpoints without formal multiplicity adjustment, so both type I and type II errors were probable. Taken together, these factors argue for cautious interpretation of the results and underscore the need for prospective, standardized, preferably randomized studies stratified by flap type and disease category.

## Conclusions

This study examined the impact of ciNPT on postoperative complications in head and neck reconstruction. Despite comparable overall complication rates between the ciNPT and non‐ciNPT groups, this study highlights practical strategies for the clinical implementation of ciNPT in complex reconstruction settings. Further research is needed to confirm its efficacy in this surgical field.

## Author Contributions

**Haruyuki Hirayama**, conceptualization, methodology, investigation, data curation, writing—original draft and editing, project administration; **Katsuhiro Ishida**, conceptualization, methodology, writing—review, supervision; **Hiroki Kodama**, writing—conceptualization, writing—review; **Doruk Orgun**, methodology, writing – review; **Masaki Nukami**, investigation, data curation; **Masato Nagaoka**, writing—review, validation; **Taisuke Akutsu**, validation, formal analysis; **Soichiro Fukuzato**, validation, formal analysis; **Takeshi Miyawaki**, writing—review, supervision.

## Disclosures

### Competing interests

None.

### Funding source

None.

## Trial Registration

umin.ac.jp/ctr Identifier: UMIN000056066.

## Data Availability

The datasets generated during and/or analyzed during the current study are available from the corresponding author on reasonable request.
